# Sustained exposure to systemic endotoxin triggers chemokine induction in the brain followed by a rapid influx of leukocytes

**DOI:** 10.1186/s12974-020-01759-8

**Published:** 2020-03-25

**Authors:** Carolyn A. Thomson, Alison McColl, Gerard J. Graham, Jonathan Cavanagh

**Affiliations:** 1 Department of Physiology and Pharmacology, Snyder Institute for Chronic Diseases, Cumming School of Medicine, University of Calgary, Calgary, Alberta Canada; 2 Institute of Infection, Immunity & Inflammation, Collage of Medical and Veterinary Life Sciences, University of Glasgow, Glasgow, UK; 3 Institute of Health & Wellbeing, Collage of Medical and Veterinary Life Sciences, University of Glasgow, Glasgow, UK

**Keywords:** Chemokines, Lipopolysaccharide, Neuroinflammation, Leukocyte recruitment, Immune-to-brain communication, Neuroimmune communication, Psychoneuroimmunology, TLR ligation

## Abstract

**Background:**

Recent years have seen an explosion of research pertaining to biological psychiatry, yet despite subsequent advances in our understanding of neuroimmune communication pathways, how the brain senses and responds to peripheral inflammation remains poorly understood. A better understanding of these pathways may be important for generating novel therapeutics to treat many patients with chronic inflammatory diseases who also suffer from neuropsychiatric comorbidities. Here we have systematically assessed the leukocyte infiltrate to the brain following systemic endotoxin exposure to better understand this novel route of neuroimmune communication.

**Methods:**

Mice were injected intraperitoneally with LPS daily for 2, 5 or 7 consecutive days. We systematically interrogated the subsequent induction of chemokine transcription in the brain using TaqMan low-density arrays. A combination of flow cytometry and immunohistochemistry was then used to characterise the accompanying leukocyte infiltrate.

**Results:**

Repeated LPS challenges resulted in prolonged activation of brain-resident microglia, coupled with an increased local transcription of numerous chemokines. After 2 days of administering LPS, there was a marked increase in the expression of the neutrophil chemoattractants CXCL1 and CXCL2; the monocyte chemoattractants CCL2, CCL5, CCL7 and CCL8; and the lymphocyte chemoattractants CXCL9, CXCL10 and CXCL16. In a number of cases, this response was sustained for several days. Chemokine induction was associated with a transient recruitment of neutrophils and monocytes to the brain, coupled with a sustained accumulation of macrophages, CD8+ T cells, NK cells and NKT cells. Strikingly, neutrophils, monocytes and T cells appeared to extravasate from the vasculature and/or CSF to infiltrate the brain parenchyma.

**Conclusions:**

Prolonged exposure to a peripheral inflammatory stimulus triggers the recruitment of myeloid cells and lymphocytes to the brain. By altering the inflammatory or metabolic milieu of the brain, this novel method of immune-to-brain communication may have profound implications for patients with chronic inflammatory diseases, potentially leading to neuropsychiatric comorbidities.

## Background

Patients with chronic inflammatory diseases such as rheumatoid arthritis, inflammatory bowel disease or psoriasis are often further burdened with neuropsychiatric symptoms such as depression, anxiety and fatigue [1–5]. These comorbidities appear to be predominantly mediated by inflammatory cytokines, which can contribute to reduced hippocampal neurogenesis, increased activation of the hypothalamic-pituitary-adrenal axis and increased glial cell reactivity and neuron excitability, together with altered tryptophan metabolism [6–9]. In keeping with this cytokine-mediated depression hypothesis, treating patients with the soluble TNFα receptor etanercept significantly ameliorated depression in patients with moderate to severe psoriasis, prior to improvements to disease severity [3]. Moreover, treating patients with type I IFNs can result in depression as a major side effect [10]; one which can even recur long after treatment stops [11]. Cytokine-mediated behavioural changes have also been reported in animal models. For example, injecting mice with lipopolysaccharide (LPS) induces a host of sickness and depression-like behaviours [6, 12], mostly attributed to the central induction of inflammatory cytokines such as IL-1β, TNFα and type I IFNs [6, 13]. However, despite an abundance of causal links between inflammatory cytokines and neuropsychiatric symptoms, we have much to learn about how the brain senses and responds to peripheral immune challenges.

Neuroimmune communication is multifactorial and has been reviewed extensively elsewhere [14]. In addition to inflammation-mediated stimulation of neuronal circuits in the periphery, and humoral routes of communication whereby inflammatory cytokines can stimulate the blood-brain barrier (BBB), perivascular macrophages or microglia within circumventricular organs, recent publications suggest that leukocytes can be recruited to the brain even in the absence of overt inflammation in the central nervous system (CNS) itself [15–17]. Presumably driven by chemokines, this phenomenon could serve as an additional and mostly overlooked route in which a systemic immune response could influence brain function.

We have previously shown that continuous LPS exposure in the periphery resulted in a dampening of inflammatory gene expression by peripheral blood leukocytes, consistent with endotoxin tolerance, but sustained expression of *Il1b*, *Tnfa* and *Cxcl10* in the brain [13]. Using a similar model, He et al. demonstrated the induction of several chemokine transcripts in the brain 12 h after a systemic LPS injection [17]. Crucially, after three daily LPS injections, the authors observed an accumulation of NK cells and neutrophils in the brain [17]. However, it has yet to be established whether these cells cross the BBB or blood-CSF barrier to enter the brain parenchyma, or LPS merely results in enhanced leukocyte marginalisation to the vasculature as has been reported previously following a single systemic LPS injection [18, 19]. It is also not clear what other leukocyte populations may also be recruited. Here we have built on these findings. Using unbiased approaches, we have systematically characterised chemokine induction and leukocyte recruitment in the brain over the course of 7 days following daily LPS injections. We show that continuous systemic LPS exposure induces the expression of multiple chemokines in the brain and drives the recruitment of various populations of myeloid cells and lymphocytes to the brain parenchyma, even after the peripheral inflammatory response to LPS has subsided. By altering the central inflammatory milieu, this influx of leukocytes may have the potential to drastically impact brain homeostasis resulting in altered mood and behaviour.

## Materials and methods

### Mice

Wild-type C57Bl/6 mice were purchased from Harlan Laboratories (Indianapolis, IN) and maintained in standard caging under specific pathogen-free conditions in the Central Research Facility, University of Glasgow. Following at least 1 week of acclimatisation, mice were injected intraperitoneally (i.p.) with 50 μg LPS derived from *Escherichia coli* serotype 055:B5 (Sigma Aldrich, MO, USA), or an equivalent volume of PBS, daily for up to 7 days. Mice were euthanised by CO_2_ exposure and perfused for 5 min with 20 ml PBS before tissue harvest. All experiments were performed on 8-week-old male mice under the auspices of UK Home Office licences.

### Preparation of single-cell suspensions

Single-cell suspensions were generated from the brain tissue by enzymatic digestion and the removal of myelin as described previously [15]. Briefly, the brains were excised from perfused mice, finely minced using a surgical blade and digested in HBSS containing 6 μg/ml Liberase TM, 5 U/ml DNase I and 25 mM HEPES (all from Sigma Aldrich, St Louis, MO) for 45 min at 37 °C. Digests were then passed through a 70-μm cell strainer and washed twice in FACS buffer (PBS containing 2 mM EDTA (Sigma Aldrich, St Louis, MO) and 10% foetal calf serum (FCS - Thermo Fisher Scientific, Waltham, MA)). Myelin was removed from the samples using myelin removal beads (Miltenyi, Cologne, Germany) in accordance with the manufacturer’s instructions.

To generate single-cell suspensions of peripheral blood leukocytes (PBL), the whole blood was collected from the ascending aorta, centrifuged at 400 g for 5 min and re-suspended in Red Blood Cell Lysis Buffer (Miltenyi, Cologne, Germany). After 10 min, cells were washed in FACS buffer and resuspended either in FACS buffer, for downstream flow cytometric analyses, or RLT buffer (Qiagen, Hilden, Germany) for subsequent RNA extraction and transcriptional analyses.

### Flow cytometry

Aliquots of up to 3 × 10^6^ cells were blocked using an anti-CD16/CD32 blocking antibody (BioLegend, San Diego, CA) and labelled with fluorescently conjugated antibodies in the dark as described previously (Thomson et al. 2018). The following antibodies were purchased from BioLegend: CD4-AF488 (GK1.5), CD8α-PE/Cy7 (53-6.7), CD11b-PE (M1/70), CD44-BV421 (IM7), CD45-BV510 (30-F11), CD45-PE/Cy7 (30-F11), CD62L-PE (MEL-14), CD64-APC (X54-5/7.1), F4/80-FITC (BM8), Ly6G-AF700 (1A8), MHCII-BV510 (M5/114.15.2), NK1.1-BV605 (PK136) and TCRβ-PerCP/Cy5.5 (H57-597). The following were purchased from eBioscience (San Diego, CA): CD11b-AF700 (M1/70) and Ly6C-eFluor 450 (HK1.4). Dead cells were excluded from analysis using Fixable Viability Dye eFluor 780 (eBioscience, San Diego, CA). Cells were analysed using an LSRII (BD Biosciences, San Jose, CA). Data were analysed using FlowJo v10 (Tree Star, Ashland, OR).

### ELISA

The concentrations of IL-1β, TNFα and IL-6 present in blood plasma were established using DuoSet ELISA Kits (R&D Systems, Minneapolis, MN) in accordance with manufacturer’s guidelines.

### RNA extraction and cDNA synthesis from the brain and PBL

The brains were cut in two, the meninges removed, and the right hemisphere was snap-frozen in liquid nitrogen and stored at − 80 °C until required. Under RNase-free conditions, half-brains were homogenised in 2 ml TRIzol Reagent (Thermo Fisher Scientific, Waltham, MA) using the TissueLyser LT (Qiagen, Hilden, Germany) and 5 mm Stainless Steel Beads (Qiagen, Hilden, Germany). RNA was then extracted from the homogenised tissue using the TRIzol Plus RNA Purification Kit (Thermo Fisher Scientific, Waltham, MA) in accordance with manufacturer’s instructions. Genomic DNA was eliminated from the columns using RNase-Free DNase kits (Qiagen, Hilden, Germany). PBL were isolated from the whole blood, as described above, and lysed in RLT buffer. Under RNase-free conditions, RNA was extracted using RNeasy Micro Kits (Qiagen, Hilden, Germany). RNA from the brain and PBL was reverse transcribed to cDNA either with the Quantitect Reverse Transcription Kit (Qiagen, Hilden, Germany) using random primers for downstream QPCR analyses or with the high-capacity RNA-to-cDNA kit (Thermo Fisher Scientific, Waltham, MA) for TaqMan low-density arrays.

### TaqMan low-density arrays

Chemokine transcription was assessed using custom-made TaqMan low-density array (TLDA) microfluidic cards (Applied Biosystems, Foster City, CA) as described previously [20]. Briefly, 100 μl of reaction mix, containing a 1:1 mixture of cDNA (from ~ 1 μg total RNA) in RNase-free water and 2x TaqMan Universal PCR Master Mix (Thermo Fisher Scientific, Waltham, MA), was loaded onto cards containing the primers and probesets for 32 genes. Cards were centrifuged to distribute the reaction mixture throughout the fluidic system and run on a Prism 7900HT Fast Real-Time PCR System (Thermo Fisher Scientific, Waltham, MA). Data were analysed using SDS2.2 software and RQ Manager. Relative gene expression was first normalised to the expression of housekeeping gene *Tbp*, which encodes TATA-binding protein, and then normalised to that of a calibrator selected arbitrarily from the vehicle control group. Fold change of gene expression in LPS-challenged mice compared to vehicle controls was calculated using the ^ΔΔ^C_T_ method.

### Quantitative real-time polymerase chain reactions

QPCR were performed in triplicate using 2x PerfeCTa SYBR Green Fast Mix (Quanta Biosystems, Gaithersburg, MD) as described previously [21], with reactions containing cDNA template and a 500-μM mix of forward and reverse primers. Primer sequences were designed using Primer3 Input Software (version 0.4.0) and manufactured by IDT technologies. Primer sequences are as follows: *Ccl3*: 5′-CAGCCAGGTGTCATTTTCCT-3′ and 5′-CAGGCATTCAGTTCCAGGTC-3′; *Ccl5*: 5′-CTACTGCTTTGCCTACCTCT-3′ and 5′-ACACACTTGGCGGTTCCTT-3′; *Cxcl1*: 5′-GCTTGCCTTGACCCTGAA-3′ and 5′-TGTCTTCTTTCTCCGTTACTTGG-3′; *Cxcl2*: 5′-AAGTTTGCCTTGACCCTGAA-3′ and 5′-TCTCTTTGGTTCTTCCGTTG-3′; *Ccr1*: 5′-GCCCTCATTTCCCCTTCAA-3′ and 5′-CGGCTTTGACCTTCTTCTCA-3′; *Ccr3*: 5′-GATTGCCTACACCCACTGCT-3′ and 5′-CGGAACCTCTCACCAACAA-3′; *Ccr5*: 5′-TTTGTCCTGCCTTCAGACC-3′ and 5′-TTGGTGCTCTTTCCTCATCTC-3′; *Cxcr2*: 5′-TGTCTGCTCCCTTCCATCTT-3′ and 5′-CCATTTCCTCTCCTCCACCT-3′; *Cxcr3*: 5′-AGTGCTTGTCCTCCTTGTAGTTG-3′ and 5′-GGTGTTGTCCTTGTTGCTGA-3′ and *Tbp*: 5′-TGCTGTTGGTGATTGTTGGT-3′ and 5′-AACTGGCTTGTGTGGGAAAG-3′. QPCR reactions were run for 40 cycles on a Prism 7900HT Fast Real-Time PCR system (Thermo Fisher Scientific, Waltham, MA). Gene expression data were first normalised to the expression of *Tbp*. Fold change values were calculated by comparing the normalised gene expression values of each sample to the mean expression level of the control group using the ^ΔΔ^C_T_ method [21].

### Immunohistochemistry

The brains extracted from LPS-treated and naïve control mice were formalin-fixed and embedded in paraffin. The brains were sliced using a precision adult brain slicer matrix to allow for the consistent histological analysis of coronal brain sections from specific regions of the brain (frontal lobe, parietal-temporal lobes and cerebellum) that were highly comparable between samples. Immunohistochemistry was performed on brain tissue sections by the Veterinary Diagnostic Service Facility at the University of Glasgow. Slides were stained with antibodies specific to CD3ε (SP7) (Vector Laboratories, Burlingame, CA), S100A9/calprotectin (MAC387) and myeloperoxidase (GA511) (Dako, Jena, Germany). For leukocyte quantification, immunoreactive cells were counted blind from three coronal sections per mouse, scanned at ×40 magnification. Cells were considered to be associated with the blood vessels or meninges if they appeared within < 1 mm of either structure.

### Statistical analysis

Data were analysed using Prism 6 software (GraphPad, San Diego, CA). Statistical tests used are included in the figure legends.

## Results

### Daily intraperitoneal injections of LPS dampen the peripheral inflammatory milieu

Using a previously published model of chronic LPS exposure [13], mice were injected intraperitoneally (i.p.) with 50 μg LPS every 24 h for up to 7 days (Supplementary Figure 1A-B). This triggered a significant drop in body weight and a substantial and sustained increase in the proportions of neutrophils and monocytes present in the circulation that persisted throughout the time period (Supplementary Figure 1B-D). Six hours following a single LPS injection, both IL-1β and IL-6 were significantly elevated in the circulation (Supplementary Figure 1E), but no TNFα was detected at this time point, consistent with previous reports [13, 22]. At later time points, neither IL-1β nor TNFα could be detected in the circulation following recurrent injections of LPS, despite the continued presence of increased numbers of monocytes and neutrophils that can produce these cytokines in response to LPS [23]. Although the level of IL-6 remained significantly elevated in the circulation of LPS challenged mice on day 2, this returned to baseline at later times (Supplementary Figure 1E). These data are in keeping with previous reports that recurrent systemic LPS challenges result in a dampening of the peripheral inflammatory milieu [13, 24].

### Daily LPS challenge triggers a sustained induction of inflammatory chemokines in the brain

**Fig. 1:**
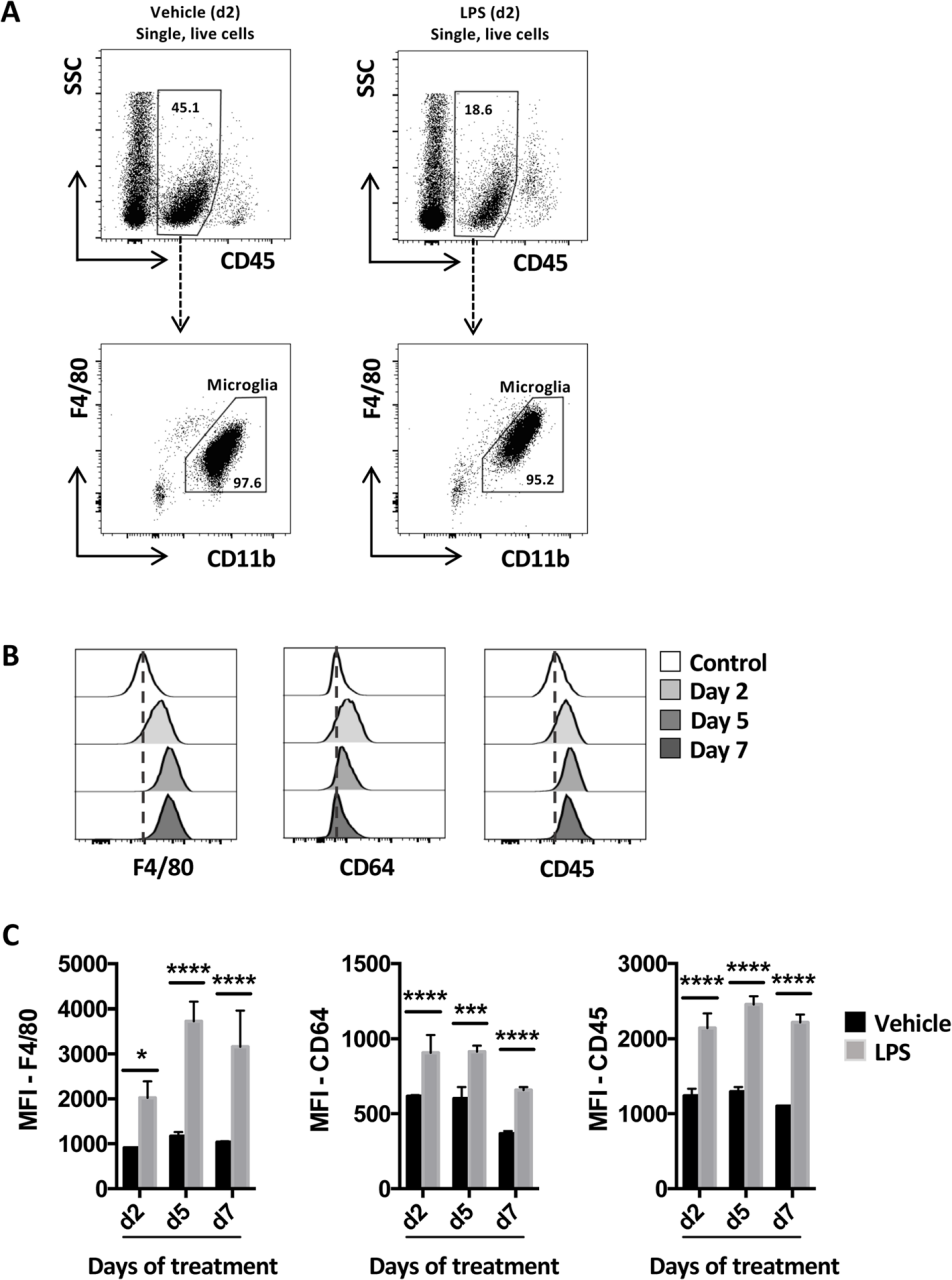
Daily systemic LPS challenges increased microglial reactivity. Mice were injected daily with LPS or vehicle (PBS) i.p. for 2, 5 or 7 days and sacrificed 24 h following the final injection. **a** Flow cytometry gating strategy and representative flow cytometry plots showing CD45^int^ microglia in the brain of vehicle (left panels) or LPS (right panels) injected mice. **b** Representative histograms and **c** quantification of the mean fluorescence intensity (MFI) of CD45, F4/80 and CD64 expression by CD45^int^ microglia in the brains of mice injected with LPS for 2, 5 or 7 consecutive days, compared with mice injected with vehicle. A control sample from the vehicle-injected day 2 group was used for comparison in **b**. Data show means ± 1SD. Significance was calculated using two-way ANOVA: *p ≤ 0.05, ***p ≤ 0.001, ****p ≤ 0.0001; *n* = 3 (vehicle), *n* = 5 (LPS). Data are representative of two independent experiments

**Fig. 2:**
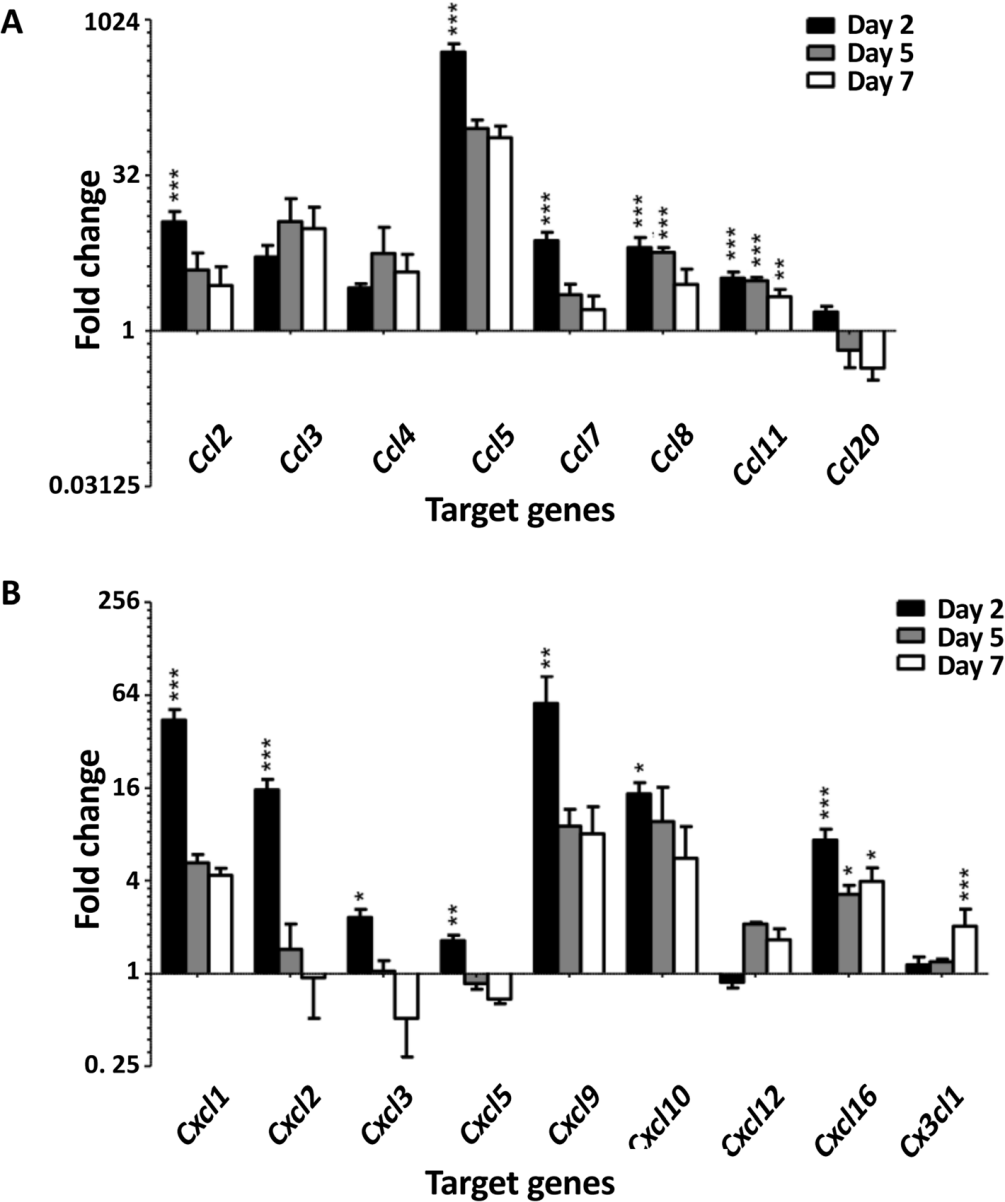
Chemokine transcription was induced in the brain in response to systemic LPS challenges. Relative expression of **a** genes encoding CC chemokines and **b** genes encoding CXC and CX_3_C chemokines in the brains of mice challenged daily with LPS for 2, 5 and 7 days compared with vehicle-injected controls. Gene expression analysis was performed using TaqMan low-density arrays and normalised to *Tbp*. Fold change was calculated by comparing normalised expression of each gene to that of a calibrator from the vehicle-injected control group using the ΔΔC_T_ method. Data show means ± 1SEM. Significance was calculated using two-way ANOVA: *p ≤ 0.05, **p ≤ 0.01, ***p ≤ 0.001; *n* = 5 per group

Flow cytometric analysis showed that microglia in the brains of LPS-challenged mice acquired a reactive phenotype [25], as indicated by increased expression of CD45, F4/80 and CD64 (Fig. 1a–c). Despite the lack of circulating IL-1β, TNFα and IL-6 at days 5 and 7, microglia remained activated throughout the model. This was associated with elevated expression of genes encoding a number of chemokines in the brain, as indicated by TLDA assays and validation of the most highly expressed transcripts using QPCR (Fig. 2a, b, Supplementary Figure 2A). On day 2, *Ccl2*, *Ccl5*, *Ccl7*, *Ccl8* and *Ccl11* were significantly upregulated in the brains of LPS-challenged mice compared with brains from PBS injected control mice, as were *Cxcl1*, *Cxcl2*, *Cxcl3*, *Cxcl5*, *Cxcl9*, *Cxcl10* and *Cxcl16* (Fig. 2a, b, Supplementary Figure 2A). By day 5, most chemokine transcripts began returning to baseline levels, but *Ccl8*, *Ccl11* and *Cxcl16. Ccl11* and *Cxcl16* remained increased until the end of the experiment on day 7. Interestingly, QPCR analysis showed that the induction of *Ccl3*, *Ccl5*, *Cxcl1* and *Cxcl2* expression in the brain was not mirrored by similar changes in peripheral blood leukocytes (PBL) (Supplementary Figure 2A), as has been previously described for *Cxcl1*0, suggesting that chemokine transcription may be regulated independently in the brain.

We next assessed the expression in the brain of genes encoding the CCR1, CCR3, CCR5, CXCR2 and CXCR3 receptors that account for much of the binding of the chemokine ligands we found to be upregulated after LPS injection. On day 2, transcript levels of *Ccr1*, *Cxcr2* and *Cxcr3* were significantly elevated in brain tissue by ~ 6.7-fold, ~ 46.6-fold and ~ 1.8-fold, respectively (Supplementary Figure 2B). By day 5, transcript levels of *Ccr1* and *Cxcr2* had begun to return to baseline, whereas *Cxcr3* transcript levels remained elevated after 7 days of daily LPS exposure. No significant increase in *Ccr3* or *Ccr5* mRNA was observed at any of the time points analysed, although there was a trend towards an upregulation of both chemokine receptors at days 5 and 7. Multiple LPS injections had no significant impact on the transcript levels of either *Cxcr2* or *Cxcr3* in PBL at any time and although *Ccr1* and *Ccr5* mRNA levels were significantly elevated in PBL at day 2, these had returned to baseline by day 5 (Supplementary Figure 2B). Therefore, chemokine receptor transcripts were either independently regulated in the brain or were differentially represented following the local recruitment of chemokine-receptor bearing leukocytes to the brain.

### Repeated systemic LPS challenges trigger a robust recruitment of leukocytes to the brain

To test whether the brain-specific increase in *Ccr1*, *Cxcr2* and/or *Cxcr3* transcripts was a downstream effect of leukocyte recruitment to the brain, we used flow cytometry to look for infiltrating leukocytes and identified as CD45^hi^ to distinguish them from brain-resident CD45^int^ microglia (Fig. 1c; Supplementary Figure 3). After 2 days of administering LPS, there was a highly significant increase in both the proportion of CD45^hi^ cells (7.2-fold) and CD45 transcript levels (3.7-fold) in the brain compared with PBS-treated controls (Supplementary Figure 3B-D). The proportion of CD45^hi^ cells remained increased on days 5 and 7 (3.9-fold and 2.7-fold, respectively), although these later changes did not attain statistical significance. Due to variations in the cellularity of single-cell suspensions of the brain following myelin removal, we were unable to accurately calculate absolute numbers.

**Fig. 3:**
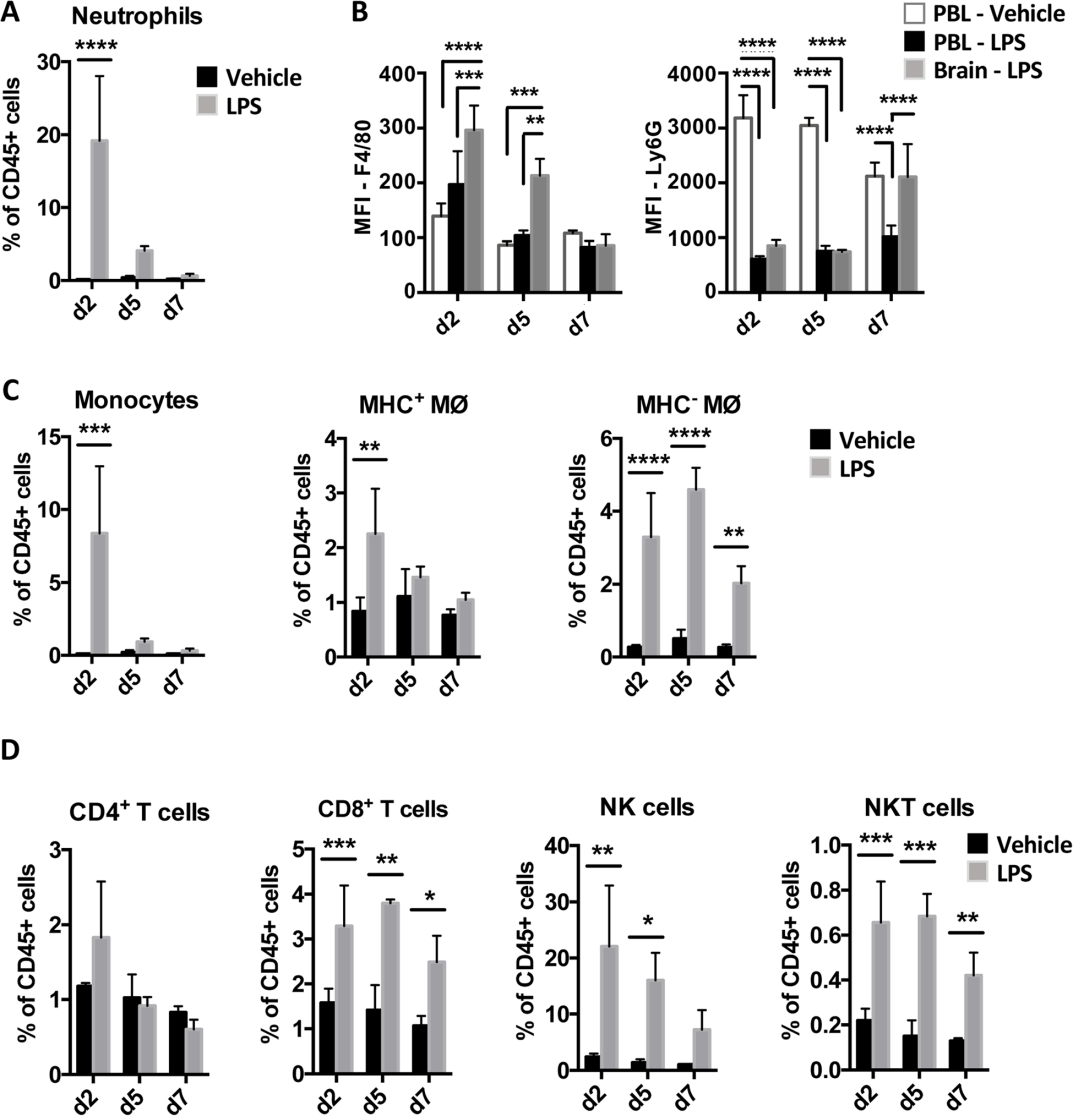
Myeloid cells and lymphocytes were recruited to the brain following peripheral LPS exposure. Recruitment of CD45^hi^ leukocytes to the brain after daily injections of LPS or vehicle for 2, 5 and 7 days as assessed by flow cytometry. **a** Percentage of CD11b^+^Ly6G^+^Ly6C^int^ neutrophils as a proportion of all single, live, CD45^+^ cells in the brain. **b** Mean fluorescence intensity of F4/80 and Ly6G expression by neutrophils in the brains of LPS-challenged mice compared with neutrophils in the blood of vehicle and LPS-injected mice. **c** Percentage of CD11b^+^Ly6G^−^Ly6C^+^ monocytes, CD11b^+^F4/80^+^Ly6C^−^ macrophages and **d** CD4^+^ T cells, CD8^+^ T cells, NK1.1^+^TCRβ^−^ NK cells and NK1.1^+^TCRβ^+^ NKT cells as a proportion of all single, live, CD45^+^ cells in the brain. For gating strategy, see Supplementary Figure 4. Data show means ± 1SD. Significance was calculated using two-way ANOVA: *p ≤ 0.05, **p ≤ 0.01, ***p ≤ 0.001, ****p ≤ 0.0001; *n* = 3 (vehicle), *n* = 5 (LPS). Data are representative of two independent experiments

CXCL1 and CXCL2 are known neutrophil chemoattractants, and the substantial upregulation of their transcription in the brain (~ 41.2-fold and ~ 114.6-fold respectively) was associated with a marked increase in CD11b^+^Ly6C^int^Ly6G^hi^ neutrophils in the brain at day 2 (Supplementary Figure 4A, Fig. 3a). This coincided with the peak of disease as assessed by weight loss and general appearance (Supplementary Figure 1B). These neutrophils were phenotypically distinct from those in the bloodstream of vehicle-injected control mice, expressing higher levels of F4/80 and lower levels of Ly6G on days 2 and 5 (Fig. 3b). Consistent with the upregulated levels of mRNA for the monocyte-attracting chemokines CCL2, CCL5, CCL7, CCL8 and CXCL3, there were also increases in the proportions of CD11b^+^Ly6C^hi^F4/80^+^ monocytes and MHCII^+^ and MHCII^−^ CD11b^+^Ly6C^−^ F4/80^+^ macrophages in the brain of LPS-injected mice at this time point (Supplementary Figure 4A, Fig. 3c). Although the numbers of monocytes and MHCII^+^ macrophages returned to baseline by day 5, the numbers of CD45^hi^MHCII^−^ macrophages remained significantly higher throughout the brain at all times after LPS injections (Fig. 3c).

The brain-specific upregulation of *Ccl3*, *Ccl5, Cxcl9* and *Cxcl10*, together with increased levels of *Ccr1* and *Cxcr3* mRNA, prompted us to examine whether lymphocytes were also recruited to the brain in response to LPS. Indeed, there were significant increases in CD8^+^ T cells, NK cells and NKT cells in the brains of LPS-challenged mice throughout the experiment, although the numbers of CD4^+^ cells did not change (Supplementary Figure 4B, Fig. 3d). T cells in the brain were almost exclusively effector memory cells, due to their high expression of CD44 and absence of CD62L expression (Supplementary Figure 4B). Thus, the enhanced early transcription of chemoattractants in the brain induced by LPS was accompanied by transient recruitment of myeloid cells to the brain, together with more sustained accumulation of lymphocytes.

### Recruited leukocytes infiltrate the brain parenchyma

**Fig. 4:**
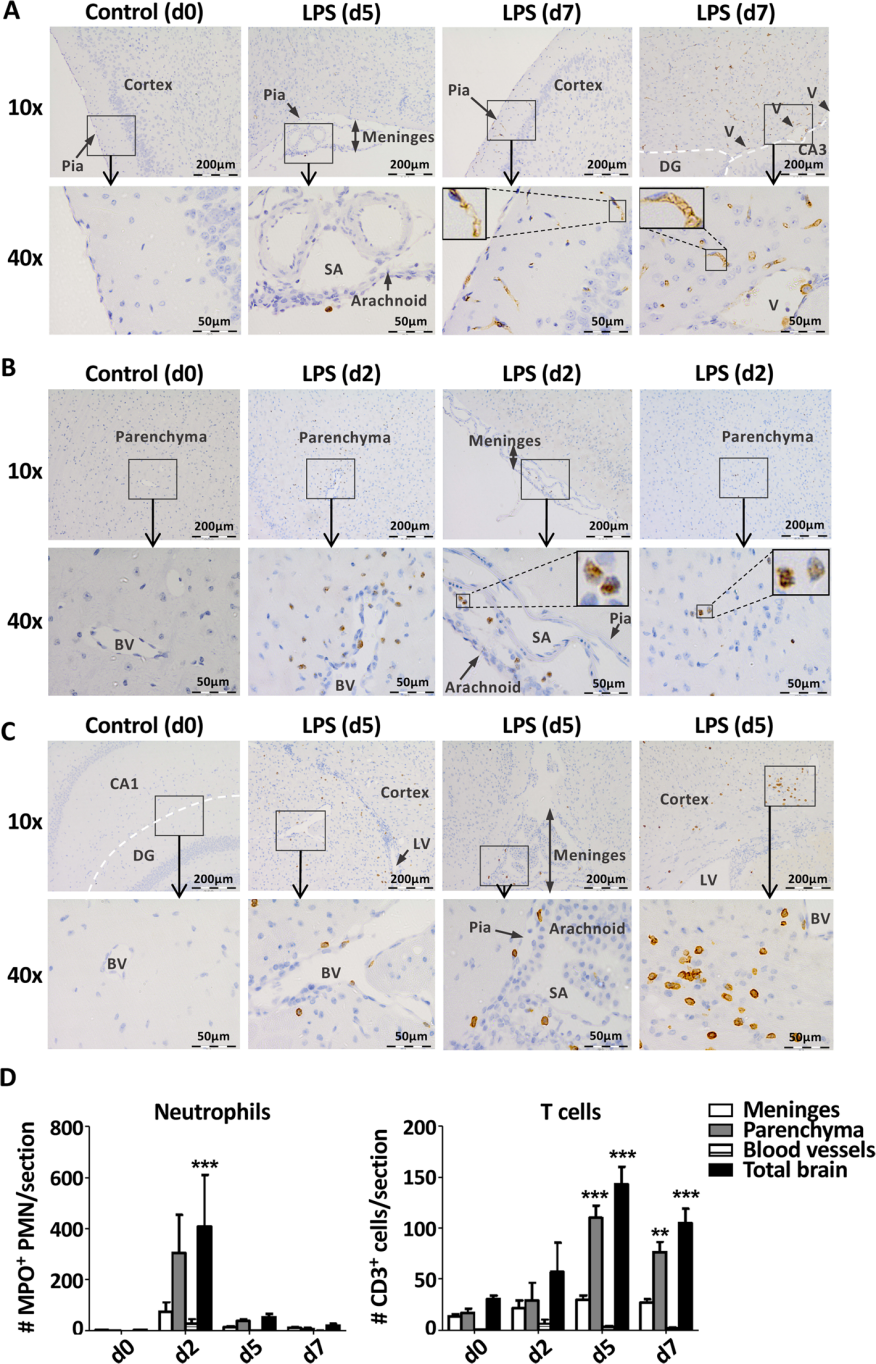
Leukocytes were recruited to the vasculature, meninges and brain parenchyma. Leukocyte distribution throughout the brain following daily injections of LPS for 2, 5 or 7 days. **a** Representative images showing CALP^+^ cells in the meninges at day 5, and amongst the cortex, ventricular space (V) and surrounding parenchyma at day 7. No CALP^+^ cells could be seen in untreated control mice (day 0). SA, sub-arachnoid space; CA3, hippocampal region; DG, dentate gyrus. **b** MPO^+^ cells in the blood vessels (BV), the meninges and the parenchyma at day 5 compared to control mice (day 0). **c** CD3^+^ cells in BV, meninges, lateral ventricle (LV) and surrounding cerebral cortex at day 5 compared to control mice (day 0). CA1, hippocampal region. **d** Quantification of MPO^+^ polymorphonuclear cells (left) and CD3^+^ T cells in meninges, parenchyma, blood vessels and whole brain. Data are presented as number of cells per coronal section +/− SEM. Three coronal sections from equivalent brain regions were analysed per sample. Significance was calculated using two-way ANOVA: **p ≤ 0.01, ***p ≤ 0.001 compared to control (day 0). *n* = 3/group

Previous studies have shown an increase in the rolling and tethering of neutrophils to the luminal surface of the BBB following acute systemic exposure to LPS [18, 19]. To establish whether the increase in leukocyte populations in the brain we found after more chronic exposure to LPS also reflected enhanced marginalisation to the vasculature, we stained tissue sections with antibodies specific for the inflammatory myeloid cell markers calprotectin (CALP) (Fig. 4a) and myeloperoxidase (MPO) (Fig. 4b). Although both of these markers can be expressed by neutrophils, monocytes and macrophages under different circumstances [26, 27], CALP-expressing cells were mononuclear and resembled either monocytes (adhered to the vasculature or ventricular space) or macrophages (in parenchyma) (Fig. 4a), whereas MPO expression in the brain was restricted to polymorphonuclear cells (Fig. 4b). Not only could these putative neutrophils and monocytes/macrophages be found in close proximity to the vasculature, but they were also detectable in the meninges and the ventricles and frequently appeared to be located within the parenchyma itself (Fig. 4a, b, and d). Consistent with the flow cytometry data, MPO^+^ polymorphonuclear cells were only found in the brain after 2 days of injecting LPS (Fig. 4b, d). In contrast, CALP expressing mononuclear cells were undetectable at day 2, but could occasionally be found in the meninges and in close proximity to the blood vessels on day 5. They could also be found in locations consistent with parenchyma on day 7 (Fig. 4b and data not shown).

Consistent with the flow cytometry data, CD3^+^ T lymphocytes were present throughout the brain at all times after injection of LPS, whereas they were rarely found in the control brains (Fig. 4c, d and data not shown). Although the location of T cells was consistent with the brain parenchyma, they could also be found in the meninges and associated with the blood vessels (Fig. 4c, d). Collectively, these data confirm that prolonged exposure to LPS in the periphery results in accumulation of myeloid and lymphoid cells in the meninges and in close proximity to the brain vasculature. Leukocytes could also be found frequently in locations consistent with infiltration of the brain parenchyma itself.

## Discussion

**Fig. 5:**
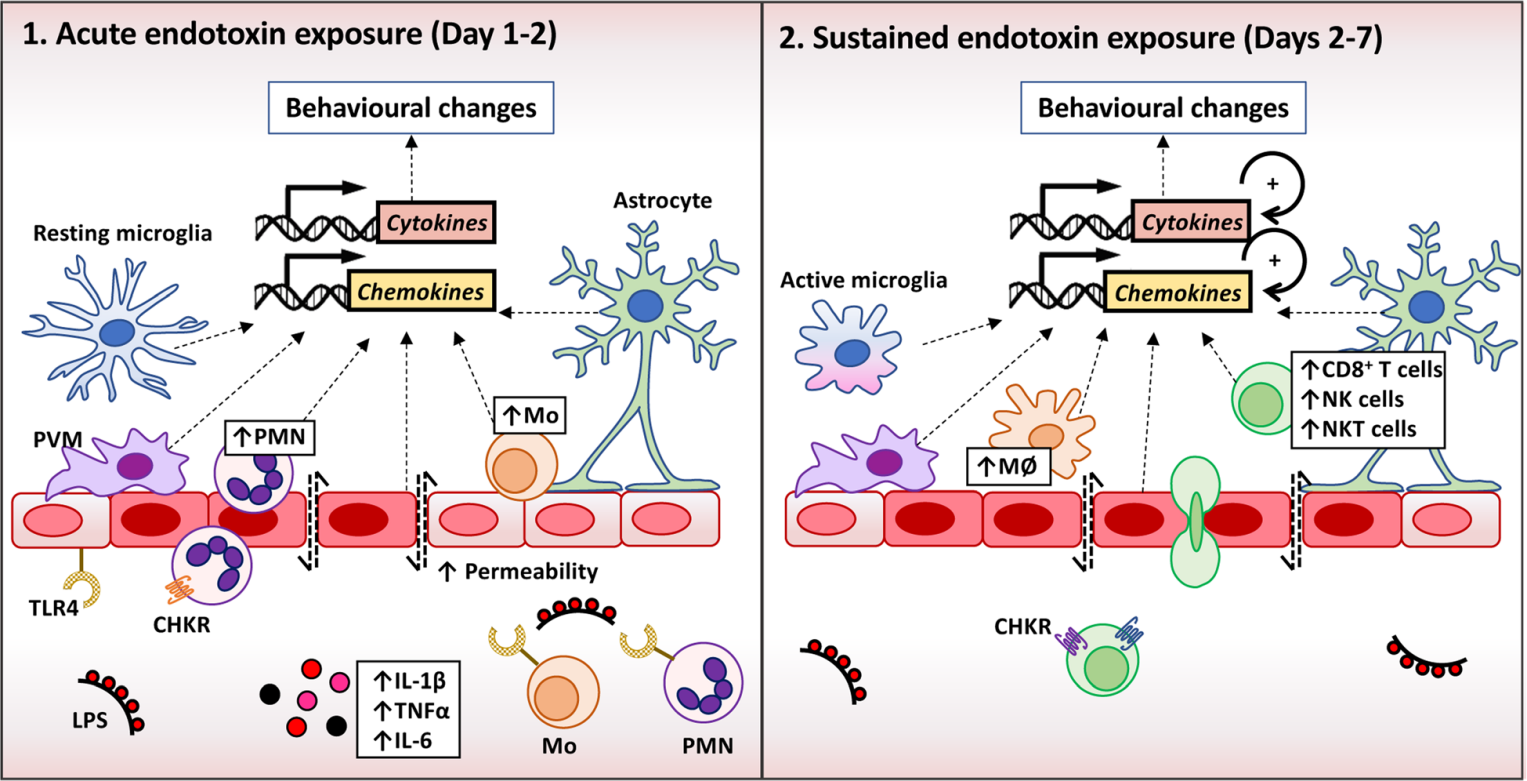
The response of the brain to acute and prolonged endotoxin exposure. (1) A single systemic challenge with LPS activates peripheral innate immune cells such as monocytes (Mo) and neutrophils (PMN) via TLR4. This triggers the release of inflammatory cytokines into the circulation, activating the cerebral endothelium and perivascular macrophages (PVM) in the circumventricular organs, which then relay inflammatory signals to the brain. Following 2 consecutive challenges, inflammatory cytokine and chemokine transcripts are upregulated in the brain. This leads to a leaky blood-brain barrier and a transient recruitment of neutrophils and monocytes to the vasculature and brain parenchyma. (2) After repeated systemic exposure to LPS, endotoxin tolerance means there is little response in the peripheral immune system, but this does not appear to occur in the brain itself [13], where there continues to be increased transcription of inflammatory cytokines and chemokines. This leads to persistent infiltration by macrophages (MØ), T cells, NK cells and NK T cells. By amplifying local inflammatory responses, this sustained recruitment of peripheral leukocytes to the brain will potentiate LPS-mediated behavioural changes

Here we have demonstrated that repeated intraperitoneal LPS challenges trigger a strong transcriptional upregulation of neutrophil chemoattractants CXCL1, CXCL2 and CXCL5 in the brain, along with enhanced expression of various chemokines involved in attracting monocytes (e.g. CCL2, CCL3, CCL5, CCL7, CCL8, CXCL3), T cells (e.g. CCL5, CXCL9, CXCL10) and NK cells (e.g. CXCL9, CXCL10, CXCL16). This was coupled with a transient influx of neutrophils and monocytes, and a more sustained presence of macrophages, CD8 T cells, NK cells and NK T cells in the brain throughout the model (Fig. 5). The temporal pattern of leukocyte recruitment mimics a classic anti-bacterial or anti-viral response, with innate cells being the first responders, triggering enhanced lymphocyte-mediated immune surveillance.

By demonstrating that long-term peripheral LPS administration induces the robust and sustained transcriptional upregulation of a wide range of chemokines in the brain, this study extends our previous findings that inflammatory cytokines and interferon-stimulated genes are upregulated specifically in the brain in the same model [13]. It also builds on findings by He et al. showing the induction of an almost identical panel of chemokine transcripts 12 h following a single peripheral LPS injection [17]. Furthermore, others using less comprehensive approaches have reported elevated levels of CCL2, CCL3 and CCL5 in the brain but not the serum following multiple LPS challenges within a 24-h period [28]. In keeping with peripheral LPS tolerance having developed in our protocol, the pattern of response we found here in the brain was distinct from that of peripheral blood leukocytes, both in terms of magnitude and duration; crucially it was also associated with an accumulation of myeloid cells and lymphocytes in the brain. While the leukocytes appeared to be situated throughout the parenchyma, they could also be detected in the meninges, the ventricles and near to the vasculature. Additional imaging techniques will be required to determine whether these cells are located within the blood vessels themselves or are simply in close proximity to the vasculature. Another limitation of this study is the unbiased approach taken, using the whole brain RNA to compare chemokine transcription in the brains of vehicle- and LPS-challenged mice. While we are able to clarify that chemokines are induced in the brain, at least at a transcriptional level, further work using immunostaining and/or fluorescent in situ hybridisation would be required to establish the anatomical location and cellular sources of these chemokines and to confirm that our transcriptional data reflects the inflammatory protein milieu in the brain.

The route and mechanisms by which leukocytes enter the brain parenchyma in the response to a peripheral inflammatory stimulus remain unclear. Several groups have shown that chemokine induction in the brain can induce leukocyte infiltration, both under neuroinflammatory and neurodegenerative disorders [29–31], in response to peripheral inflammation [15–17] and during CNS viral infection [20, 32, 33]. Chemokines are known to trigger integrin-mediated binding of immune cells to vascular surfaces [34, 35], playing a key role in the trafficking of immune cells between meningeal blood vessels, the meninges and CSF [36], and extravasation across the BBB [37]. As such, it seems likely that the production of chemokines in the brain allows leukocyte populations to migrate across the blood vessels and enter the CSF and/or brain parenchyma. This would be consistent with our findings that neutrophils, monocytes and T cells could all be detected in the meninges following repeated administration of LPS. We also observed neutrophils and T cells in close proximity to blood vessels and ventricles. However, more often they appeared to be distributed throughout the parenchyma and not closely associated with the vasculature. Leukocyte recruitment to the brain is possibly aided by impaired vascular integrity, as has been described following a single dose of LPS [17, 38]. While we cannot rule out the possibility that local proliferation of brain-resident macrophages and patrolling memory T cells could contribute to the phenotypes described here, the healthy brain is devoid of neutrophils. Thus, the substantial increases in neutrophil numbers we observe in the brain are almost certainly recruited from the periphery.

The brain-specific chemokine induction triggered by repeated administration of LPS was paralleled by an increase in mRNA encoding CCR1 (binds CCL3, 5, 7, 14, 15, 16 and CCL23), CXCR2 (binds CXCL1-5, 7 and 8) and CXCR3 (binds CXCL9-11). There was also a trend towards an upregulation of both CCR3 (binds CCL5, 7, 11, 13 and 26) and CCR5 (binds CCL3, 4, 5 and 8) mRNA in the brain at days 5 and 7. Although some of these receptors can be expressed by neurons and glial cells [39, 40], we believe the increases in chemokine receptor transcripts we observed are due to the increased numbers of leukocytes expressing these receptors in the brain, as these two processes showed similar patterns. For example, *Cxcr2* and *Ccr1*, expressed mainly by neutrophils and monocytes respectively, were elevated at day 2 of the LPS model, at the same time as neutrophil and monocyte numbers were increased in the brain. In addition, *Cxcr3*, expressed by T cells and NK cells was elevated throughout the model, reflecting a steady influx of T cells and NK cells at all time points. However, we cannot rule out the possibility that systemic LPS challenges also result in the regulation of chemokine receptor transcripts by brain-resident neurons and glial cells. Together, these findings clearly demonstrate that leukocytes accumulate within the brain in response to systemic LPS exposure, both in the region of the blood-brain barrier level and possibly in the parenchyma itself, and that this may be mediated by inflammatory chemokine production.

NK cells have been shown to produce neutrophil-recruiting chemokines after being cocultured with microglia [17]; thus, it is possible that an early recruitment of NK cells is responsible for the subsequent accumulation of neutrophils in this model. In fact, the authors demonstrated that the recruitment of NK cells to the brain following daily peripheral LPS injections preceded that of neutrophils by a matter of hours and that depleting NK cells prevented neutrophil and monocyte accumulation in the brain [17]. Here we have extended these findings, particularly by looking at the kinetics of these responses over a longer time. We have characterised the transcriptional chemokine response in the brain in unprecedented detail over this time frame and have used a combination of flow cytometric analyses and immunohistochemistry to assess the entire leukocyte infiltrate. Not only do we demonstrate that neutrophil and monocyte accumulation is transient, but also we show the recruitment of additional leukocyte populations, including macrophages, CD8^+^ T cells and NK cells, and crucially, our data appear to show the presence of leukocyte populations within the brain parenchyma. The extent of leukocyte distribution throughout the brain is a novel and surprising finding, particularly as others have only noted the presence of leukocytes within the cerebral vasculature [18, 19, 41]. The exact location of the infiltrating cells will require further studies using 3-dimensional imaging techniques.

Leukocyte recruitment to the brain after repeated peripheral administration of a TLR ligand may seem counterintuitive, particularly when this protocol leads to tolerance in the periphery. However, this process may be one way in which the brain prepares to protect itself against potential invasion by an organism present elsewhere in the body. Nevertheless, cells such as neutrophils and Ly6C^hi^ monocytes are also highly associated with causing bystander tissue damage, making this seem a risky reaction in such a fragile tissue [42–44]. One possible explanation could be that some of these recruited cells represent the heterogeneous population collectively termed myeloid suppressor cells (MDSC), which show similarities to both neutrophils and monocytes and which can have anti-inflammatory properties, including suppression of T cell functions [45]. This is supported by the cell surface phenotype of the neutrophils we found in the brain, which had a more immature phenotype than steady-state blood neutrophils, a phenotype associated with MDSC [46]. Another possibility is that recently recruited Ly6C^hi^ monocytes differentiate into tissue-resident macrophages, including microglia, during the resolution of inflammation, again offering the potential for tissue protection and/or repair [47]. This has been described in Alzheimer’s disease, during which recruited monocytes differentiate into microglial-like cells and aid in the clearance of amyloid plaques [48]. Thus, the recruitment of immature monocytes and neutrophils to the brain of LPS treated mice may actually reflect an attempt to protect the brain from tissue damage during the systemic inflammatory response. Paradoxically, at least some of the infiltrating mononuclear cells expressed the inflammatory marker calprotectin. Although monocyte and macrophage recruitment to the brain was greatest at days 2 and 5 of the LPS model, calprotectin reactivity could not be detected in the brain until day 7. Transcriptional profiling data shows that while calprotectin is expressed by bone-marrow monocytes, it is not expressed by circulating blood monocytes (Immunological Genome Project [49]), suggesting that it may be induced on monocytes and macrophages after they have been exposed to the environmental milieu of certain tissues, in this case, the brain. This is supported by the fact that monocytes arriving in the normal colon do not upregulate calprotectin until they are partway through the process of differentiation into macrophages (Prof Allan Mowat, personal communication). As calprotectin can amplify macrophage- and neutrophil-mediated inflammatory responses [50, 51], the recruitment of calprotectin-expressing macrophages may have a detrimental effect on the brain.

The infiltration of the brain by myeloid cells was accompanied by sustained accumulation of CD8^+^ T cells, NK cells and NKT cells. Infiltrating T cells were almost exclusively CD62L^−^CD44^hi^, consistent with the dogma that only activated T cells can enter the brain parenchyma [52]. The high proportion of lymphocytes, particularly CD8^+^ T cells, present in the brain following systemic LPS challenge was a novel and surprising finding. Each of these populations is known to be strong producers of IFNγ [53, 54], which can have a highly detrimental impact on brain function [55–57]. IFNγ is also a potent inducer of indoleamine-2,3-dioxygenase, which can mediate behavioural changes through the metabolism of tryptophan [58]. As before, the question arises of what these lymphocyte populations might be doing in a tissue where prevention of pathology would seem of paramount importance. We have shown previously that this protocol of LPS administration triggered a type-1 IFN response in the brain [13]. As both type I and type II IFNs are a classic hallmark of anti-viral immunity, these findings could again support the idea that the T cell response in the brain reflects enhanced lymphocyte-mediated immunosurveillance in anticipation of a potential viral threat.

High doses of LPS are an aggressive assault on the peripheral immune system and have been used to model sepsis [59], as well as sickness behaviour and immune-mediated depression-like behaviour in rodents [6–8]. However, it should be noted that leukocyte recruitment to the brain also occurs in response to inflammatory stimuli other than LPS and does not require such an aggressive stimulus. Indeed, we have previously reported increased expression of chemokines and infiltration of monocytes, T cells and NK cells in the brain following TLR7/8 induced skin inflammation [15]. Interestingly, these effects were not recapitulated by I.P. administration of TLR7/8 ligand, suggesting that the response of the brain to systemic inflammation may depend on both the nature and location of the stimulus. Furthermore, De Mello and colleagues demonstrated a CCR2-mediated recruitment of monocytes to the brain following hepatic inflammation [16]. Collectively, these findings add weight to the notion that leukocyte trafficking to the brain may play a key role in immune-to-brain communication that has previously been overlooked. Further experiments will be required to establish the precise contextual requirements necessary for leukocyte recruitment to the brain in response to systemic inflammation.

Neuropsychiatric symptoms, such as depression and anxiety, are common comorbidities in patients suffering from chronic inflammatory disorders, such as rheumatoid and psoriatic arthritis, multiple sclerosis and inflammatory bowel diseases [1–5]. These comorbidities represent a major burden both to patients and the health care system since they are associated with a poorer clinical outcome. The exact mechanisms underlying these effects remain to be determined with certainty. However, although it is difficult to examine biological pathways (such as leukocyte infiltration of the brain) in clinical practice, recent work shows that leukocyte recruitment to the brain vasculature and/or parenchyma may contribute to clinically relevant behavioural phenotypes in mice. For example, the production of IL-1β by monocytes adherent to the cerebral vasculature is responsible for driving anxiety behaviour in response to social stress in mice [60]. Similar processes may be involved in the long-lasting cognitive impairments that have been reported in humans 12–18 months after sepsis-associated delirium [41]. In support of this, mice infected with *Salmonella pneumonia* have increased rolling and adhesion of monocytes and neutrophils to the BBB and the associated cognitive impairments can be prevented by blocking monocyte recruitment to the vasculature [41]. Therefore, recruitment of leukocytes to the cerebral vasculature and/or the brain parenchyma may contribute to behavioural changes following sustained endotoxin exposure or in chronic inflammatory disease (Fig. 5).

Cytokines such as type-1 IFNs, IFNγ, TNFα and IL-1β can affect brain homeostasis and consequently have also been implicated in driving neuropsychiatric symptoms associated with chronic inflammation, as has altered metabolism of tryptophan via IDO [6–10]. Systemic LPS challenge is one of the most widely used models for studying the effects of peripheral immune activation on behaviour [6–8]. As well as triggering acute, cytokine-induced sickness behaviours, such as fever, anorexia and decreased motor function, it also induces more prolonged depression-like behaviours such as anhedonia and social withdrawal [6]. Repeated LPS challenge also triggers behavioural changes [61] that are likely to reflect a combination of cytokine-mediated effects and recruitment of leukocytes to the brain. We have previously reported that TLR7/8-mediated skin inflammation leads to leukocyte recruitment to the brain and this is associated with behavioural changes and reduced hippocampal neurogenesis [15]. Further studies using specific chemokine receptor-deficient mice or integrin-specific antibodies to block leukocyte recruitment will be required to determine directly whether this process causes changes in behaviour in the current model of repeated LPS challenge. Characterising the leukocyte infiltrate in the brain following different models of peripheral inflammation and understanding how this contributes to the local inflammatory or metabolic milieu could be a crucial step towards the generation of novel therapeutic strategies to ameliorate depressive symptoms in patients suffering from chronic inflammatory disorders.

## Conclusions

In summary, prolonged exposure to LPS in the periphery triggers a robust induction of chemokine transcription in the brain, accompanied by the recruitment of myeloid cells and lymphocytes to the brain parenchyma. The downstream implications of such a response remain unclear. However, by altering brain homeostasis, cytokines, chemokines and metabolites produced by infiltrating immune cells have the potential to profoundly impact brain function, mood and behaviour. This study adds weight to the notion that leukocyte recruitment to the brain may serve as a novel route of neuroimmune communication and opens the door to exploring similar phenomena using different peripheral inflammatory stimuli.

## Supplementary information

**Additional files 1: Figure S1**. Daily LPS injections triggered a robust egress of myeloid cells from the bone marrow but a subdued peripheral cytokine response. (A) Experimental model. Mice were injected with LPS or vehicle at days 0, 1, 2, 3, 4, 5 and 6, and sacrificed at 6 hours following initial injection, and at days 2, 5 and 7, 24 hours after their final injection. (B) Change in body weight was monitored throughout the model. (C) Flow cytometry gating strategy of circulating CD11b^+^Ly6G^+^Ly6C^int^ neutrophils and CD11b^+^Ly6G^-^Ly6C^+^ monocytes. (D) Percentage of circulating CD11b^+^Ly6G^+^Ly6C^int^ neutrophils and CD11b^+^Ly6G^-^Ly6C^+^ monocytes as a proportion of single, live, CD45^+^ cells in the blood. Data show means ± 1SD. *n*=5/group. Data representative of two independent experiments. (E) Plasma concentrations of IL-1β, TNFα and IL-6 at 6 hours and day 2, 5 and 7 of the LPS model. n=5-8/group. Significance was calculated using two-way ANOVA: *p≤0.05, **p≤0.01, ***p≤0.001, ****p≤0.0001.

**Additional files 2: Figure S2.** Temporal expression of chemokines and their receptors in brain and by PBL following daily LPS injections. Gene expression analysis of (A) chemokines and (B) chemokine receptors in the brain and by PBL following daily systemic LPS injections for 2, 5 and 7 days as determined by QPCR. Gene expression was normalised to that of *Tbp*. Fold change was calculated by comparing the normalised gene expression values of each sample to the mean expression level of the control group using the ^ΔΔ^C_T_ method. Significance was calculated using two-way ANOVA: *p≤0.05, **p≤0.01, ***p≤0.001, ****p≤0.0001. *n*=4/group.

**Additional files 3: Figure S3.** Proportions of CD45hi leukocytes in the brains of LPS and vehicle-treated mice. (A) Gating strategy and representative plots of single, live, CD45^+^ cells from mice injected with LPS or vehicle for 2, 5 or 7 consecutive days. (B) Relative and proportion of CD45^int^ and CD45^hi^ cells and (C) percentage of CD45^hi^ cells in the brain. The latter shown as a percentage of all single, live CD45^+^ cells. *n*=3 (vehicle) *n*=5 (LPS). (D) *Cd45* transcript abundance determined by QPCR and normalised to that of *Tbp*. Fold change was calculated by comparing the normalised gene expression values of each sample to the mean expression level of the control group using the ^ΔΔ^C_T_ method. n=4/group. Significance was calculated using two-way ANOVA: **p≤0.01, ***p≤0.001.

**Additional files 4: Figure S4.** Flow cytometry gating strategy for infiltrating leukocytes. (A) Myeloid cell gating strategy. CD11b^+^ cells were identified from single, live CD45^hi^ leukocytes in the brain. Macrophages (MØ) were further identified as being F4/80^+^Ly6C^-^ and (i) were divided into two populations based on MHC class II expression. Neutrophils were F4/80^-^Ly6C^int^ and (ii) Ly6G^+^. Monocytes were F4/80^+^Ly6C^hi^ and (iii) Ly6G^-^. (B) Lymphocyte gating strategy. Single, live CD45^hi^ leukocytes were split into NK1.1^+^TCRβ^-^ NK cells, NK1.1^+^TCRβ^+^ NKT cells and NK1.1^-^TCRβ^+^ T lymphocytes. NK1.1^-^TCRβ^+^ T lymphocytes were then further separated based on the expression of CD4 and CD8. (C) Representative plots showing expression of CD62L and CD44 by CD4^+^ and CD8^+^ T cells in the brain.

## Data Availability

All data generated or analysed during this study are included in this published article (and its supplementary information files).

## Abbreviations

ANOVA: Analysis of variance
BBB: Blood-brain barrier
CALP: Calprotectin
CNS: Central nervous system
IDO: Indoleamine 2,3-dioxygenase
LPS: Lipopolysaccharide
MDSC: Myeloid suppressor cells
MPO: Myeloperoxidase
PBL: Peripheral blood leukocytes
TLDA: TaqMan low-density array
