# Supplementary material for: Sustained exposure to systemic endotoxin triggers chemokine induction in the brain followed by a rapid influx of leukocytes
Source: J Neuroinflammation. 2020 Mar 25;17:94. doi: 10.1186/s12974-020-01759-8 (PMC7098135; doi:10.1186/s12974-020-01759-8)

## Supplementary figure 2

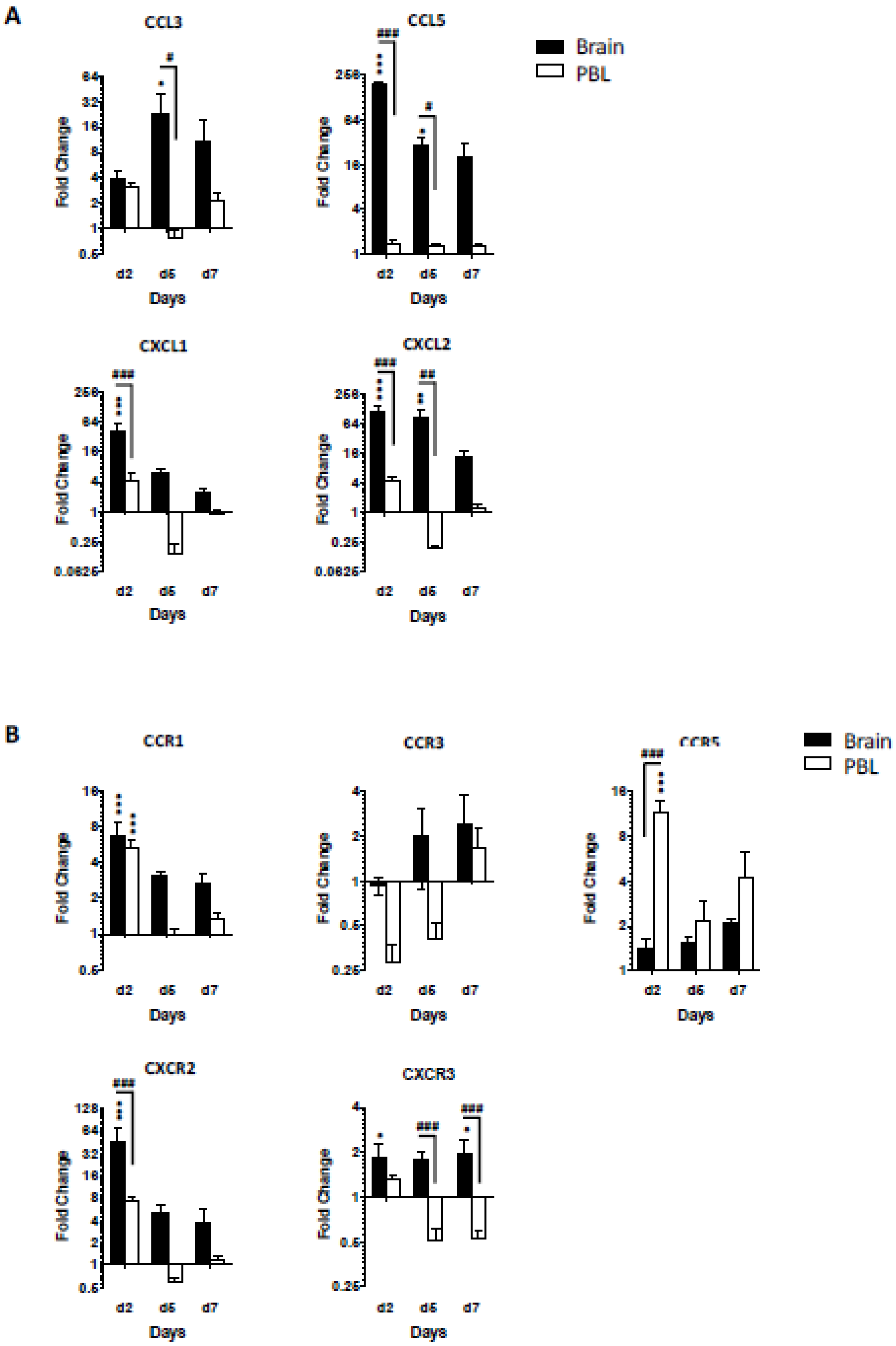

Supplement: Supplementary file 2 [file 12974_2020_1759_MOESM2_ESM.docx]
